# Porphyrin Derivative Nanoformulations for Therapy and Antiparasitic Agents

**DOI:** 10.3390/molecules25092080

**Published:** 2020-04-29

**Authors:** Daiana K. Deda, Bernardo A. Iglesias, Eduardo Alves, Koiti Araki, Celia R. S. Garcia

**Affiliations:** 1 Department of Fundamental Chemistry, Institute of Chemistry, University of Sao Paulo, Av. Prof. Lineu Prestes 748, Butanta, Sao Paulo, SP 05508-000, Brazil; daianakdn@gmail.com (D.K.D.); koiaraki@iq.usp.br (K.A.); 2 Bioinorganic and Porphyrinoid Materials Laboratory, Department of Chemistry, Federal University of Santa Maria, Av. Roraima 1000, Camobi, Santa Maria, RS 97105-900, Brazil; bernardopgq@gmail.com; 3 Department of Life Science, Imperial College London, Sir Alexander Fleming Building, South Kensington, London SW7 2AZ, UK; eduardo.alves@imperial.ac.uk; 4 Department of Clinical and Toxicological Analyses, School of Pharmaceutical Sciences, University of Sao Paulo, Av. Prof. Lineu Prestes, 580, Sao Paulo, SP 05508-900, Brazil

**Keywords:** porphyrins, nanoformulation, nanocapsules, micelles, malaria

## Abstract

Porphyrins and analogous macrocycles exhibit interesting photochemical, catalytic, and luminescence properties demonstrating high potential in the treatment of several diseases. Among them can be highlighted the possibility of application in photodynamic therapy and antimicrobial/antiparasitic PDT, for example, of malaria parasite. However, the low efficiency generally associated with their low solubility in water and bioavailability have precluded biomedical applications. Nanotechnology can provide efficient strategies to enhance bioavailability and incorporate targeted delivery properties to conventional pharmaceuticals, enhancing the effectiveness and reducing the toxicity, thus improving the adhesion to the treatment. In this way, those limitations can be overcome by using two main strategies: (1) Incorporation of hydrophilic substituents into the macrocycle ring while controlling the interaction with biological systems and (2) by including them in nanocarriers and delivery nanosystems. This review will focus on antiparasitic drugs based on porphyrin derivatives developed according to these two strategies, considering their vast and increasing applications befitting the multiple roles of these compounds in nature.

## 1. Introduction

The development of resistance to conventional drugs by pathogens is growing rapidly, decreasing their effectiveness in short time, taking out the major instrument to combat several types of diseases around the world. In fact, it is regarded as one of the most important clinical challenges to assure the public health in the future. In this context, strategies to overcome the limitations of conventional treatment modalities are being considered, but researchers have also focused their attention on the development of new, safer, potent, less invasive, and cost-effective drugs, and eventually that act by means of new mechanisms that are immune to development of resistance. Among the several classes of new drugs, porphyrins and their derivatives have gained prominence and their application on medicine has been reported for several decades.

Porphyrins are *N*-heterocyclic compounds that can be found in most biological systems, which contribute in the electron and oxygen transport and storage, as well as catalytic centers for biotransformation such as in the cytochrome P450. The macrocycle compound denominated porphine is based on 20 carbon atoms and four nitrogen atoms, characterized by the fact that four pyrrole rings are connected by methenyl bridges forming an aromatic macrocyclic compound. Furthermore, the nitrogen atoms of the four pyrrole rings are pointing inward towards the center generating a more or less rigid square, tetra-coordinating cavity, a special arrangement capable of binding the majority of metal ions forming the respective metal complexes, generating a class of compounds called metalloporphyrins when the porphine ring is substituted at the pyrrolic and/or *meso*-carbon positions [1,2]. In biological systems, metalloporphyrins have only β-pyrrolic substituents and appear conjugated to proteins, forming supramolecular structures of great importance such as hemoglobin, myoglobin, cytochromes, catalases, and peroxidases, as well as chlorophylls and bacteriochlorophylls when in reduced forms [3,4,5,6].

The interesting structural, spectroscopic, and photochemical properties, as well as diverse functions of porphyrins in biological systems, have drawn the attention of scientists around the world. However, their preparation, especially of nonsymmetrical derivatives, were shown to be quite difficult, challenging the chemists for the development of strategies for the preparation of porphyrin derivatives that could mimic such properties [7,8,9] Some examples of porphyrin-type derivatives include chlorins, benzoporphyrins, purpurins, texaphyrins, phthalocyanines, naphthalocyanines, and protoporphyrin IX [10]. In fact, the field of porphyrin synthesis evolved in such a way that almost any type of derivative can be prepared, for example, allowing the realization of complex porphyrin supramolecular structures and materials with interesting catalytic, photochemical, photophysical, and electrochemical properties, exploiting the different substituent groups attached to the periphery of the porphyrin ring as well as the contrasting electrocatalytic and photochemical properties imparted by the metal ions coordinated to the center of the macrocycle compounds. In fact, those supramolecular materials can be tailored with suitable properties, aiming application in sensors, electronic devices, catalysis, photocatalysis, and also in medicine [11,12,13,14,15,16].

Porphyrin derivatives have low dark toxicity, high tumor uptake, and absorption in the therapeutic window (600 to 800 nm) in addition to interesting photo-physical and photo-thermal properties making them interesting compounds for application in medicine, more specifically in photodynamic therapy (PDT) [17,18]. In this treatment modality, highly reactive species, such as singlet oxygen, are generated very locally by the porphyrin when light is absorbed, allowing the control of the treatment site since it depends on the presence of both the photosensitizer and light. In fact, the singlet oxygen and other reactive species can destroy microorganisms and cancer cells [19,20,21]. For example, PDT has also been applied for bacterial, fungal, parasitic, and viral infections [22,23,24,25,26,27,28,29,30], age-related macular degeneration [31], oral diseases [32,33,34,35], and in the treatment of skin diseases such as acne vulgaris, cutaneous leishmaniasis, psoriasis, and papilloma virus infections [36,37,38,39,40,41,42,43]. Another interesting property of porphyrins are their photoinduced fluorescence and phosphorescence, explored in diagnosis by fluorescence tracking and imaging. In addition, porphyrin derivatives metallated with paramagnetic transition metal ions are able to shorten the nuclear spin relaxation time of protons and can be used for diagnostics purpose by magnetic resonance image (MRI) or they can possess coordinating sites especially designed to bind radiopharmaceuticals for diagnostics by positron emission tomography/computed tomography (PET/CT) imaging [3,19].

Among the potential applications of the photochemical properties of porphyrins, they have been highly considered for the control of malaria, a disease caused by the protozoan parasite *Plasmodium falciparum* [44]. In fact, porphyrin derivatives have been investigated as alternatives for the control of the infection vectors by photodynamic antimicrobial chemotherapy (PACT) [22,45,46,47,48] and also as antimalarial drugs [49,50,51,52,53,54,55]. Porphyrins are structurally similar to hematin and are being tested against *Plasmodium* parasites since the parasites infect red blood cells and feed themselves on hemoglobin, generating protoporphyrin-IX (hematin) as toxic byproduct that is neutralized by the formation of hemozoin. Their mechanism of action is based on the prevention of formation of Fe(III)-propionate bonds, the key step in the hemozoin crystal formation, thus inhibiting the natural detoxification route [50,56,57].

Although porphyrins have demonstrated potential in the treatment of several diseases, including malaria, the low efficiency in reducing the growth of *P. falciparum* generally is associated with their low solubility in water [58] and bioavailability as well as slow diffusion through the erythrocyte membrane [52,53] that have precluded their biomedical applications. However, those limitations can be overcome by using two strategies: (1) By introducing structural modifications in the macrocycle ring increasing the hydrophilicity while enhancing their interaction with biological membranes [59,60,61,62,63,64,65] and (2) by using new strategies to improve the compatibility and delivery of the porphyrin derivatives, for example, by nanoencapsulation, protecting them from the external aqueous medium [55,66,67,68,69]. This review will focus on antiparasitic drugs based on porphyrins’ derivatives developed according to these two strategies and their applications.

## 2. General Aspects of the Porphyrin Properties

A wide variety of porphyrins and analogues have been developed and explored as powerful functional tools and as building blocks of supramolecular systems given their tunability, rich coordination chemistry, strong light absorption, and high light emission properties [70,71,72,73,74,75,76,77]. Such features conferred to these molecules a range of biological, photochemical, and photophysical properties with relevant and direct application on diseases’ treatment [19,78,79], biological imaging [19,80], and analytical methods [81,82], as well as applications in industrial [83], photocatalytic [84], molecular photovoltaics [85,86], and nonlinear optics (NLO) devices [87]. Nevertheless, it is in the field of medicine that these molecules have the highest prominence, especially in photodynamic processes such as PDT and antimicrobial photodynamic therapy (aPDT). As previously mentioned, they can generate singlet oxygen [88], a highly reactive species upon photosensitization, but ultrasound was also shown to sensitize porphyrins and this premise has been explored in sonodynamic therapy (SDT). In this therapy, porphyrin compounds, such as hematoporphyrin and protoporphyrins, can kill selective targets by generation of reactive oxygen species (ROS) as consequence of cavitation effects [89]. This technique benefits from the fact that ultrasound can penetrate deeply into tissues and can be focused on a smaller volume as compared to light [90].

The aromatic character of the porphyrin ring assures strong π→π* electronic transitions in the visible range, especially in the 400–450 nm range, where the Soret band with the highest molar absorptivity coefficient appears, followed by two or four lower intensity Q-bands in the 500–700 nm range, conferring an intense dark purple color to those tetrapyrrolic macrocycles [91] in solid state. Generally, excited-state porphyrins have higher absorption coefficients at the Q-bands, an interesting property that can be used in photobiological and photochemical processes. Their emission spectrum generally has a characteristic pattern with two emission bands in the 600 to 800 nm range, when excited in any of the absorption bands in the visible range (Soret or Q-bands). The fluorescence quantum yield (Φ_fl_) tends to be moderate to high depending on the metal ion coordinated to the ring center and the substituents. In fact, depending on the peripheral groups attached to the porphyrin ring, intersystem crossing processes can occur, increasing the population in the excited triplet state, which is fundamental for the formation of reactive oxygen species (ROS).

The excitation of porphyrins and analogue compounds results in electron density reorganization followed by either phosphorescence, fluorescence, or intersystem crossing into an excited triplet state [3]. The spin-allowed fluorescence transition enables their use as diagnostic tools in fluorescence-guided tumor dissections and imaging, while the excited triplet state generated upon intersystem crossing enables their use in PDT, aPDT, and SDT. As example, porphyrin derivatives have been used as fluorescent markers for cancer imaging since as early as the 1920s [92] and as contrast agents for MRI since the 1980s [93].The coupling of porphyrins with a variety of species with MRI contrast properties and radiopharmaceuticals generated new probes for positron emission tomography (PET) and in vivo MRI [94,95,96]. Different types of porphyrins and metalloporphyrins have been investigated for biomedical imaging applications [94], fluorescence tracking, and bio-sensors [3,97]. In general, *meso*, *meso*-aryl, and β-substituted porphyrins are commonly used in photophysical/photobiological processes, as shown below. Nonsymmetric porphyrins have been explored in nonlinear optic (NLO) materials [98], two-photons absorption (2PA) systems [99,100], molecular probes [101], and antiparasitic target molecules [55]. All these applications have benefited from surface modifications with porphyrin-based molecules, thus allowing a better control of their physico-chemical and pharmacological properties [19].

## 3. Structural Modifications Enabling Antiparasitic Activity

**Figure 1:**
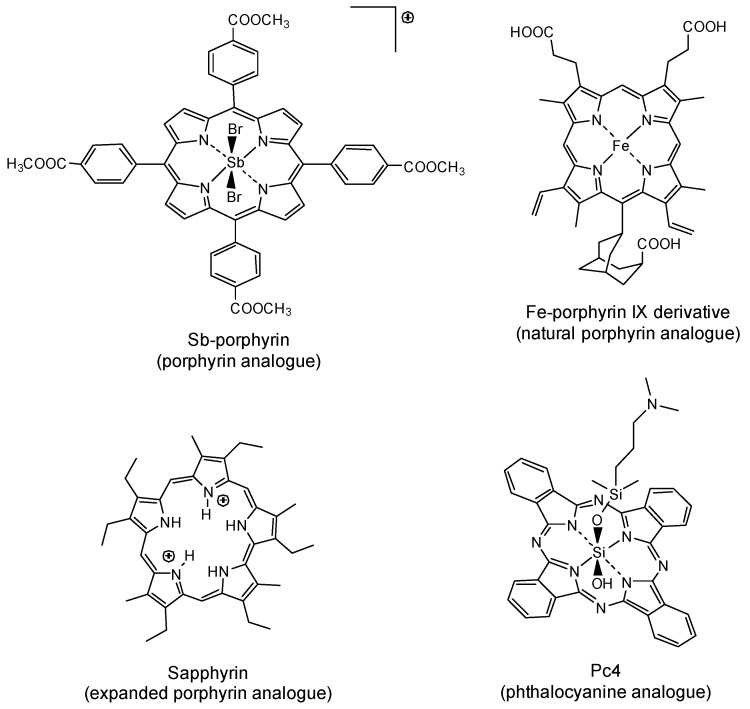
Chemical structures of the most relevant tetrapyrrolic macrocycle candidates as photosensitizers for antiparasitic application.

Although porphyrins and related macrocycles have been used as photosensitizers since the discovery of PDT due to their excellent photophysical properties, the search for ideal photosensitizer properties is still a major challenge that can be tackled by changing the structure of the peripheral substituents, thus adjusting their solubility and interaction with biological media while preserving the porphyrin core. This can be realized by taking advantage of the versatility of tetrapyrrole macrocycles to chemical transformation, producing a huge number of derivatives with unique electronic and interaction properties. Accordingly, this section will be focused on how structural modifications can improve the properties of tetrapyrrolic macrocycles (porphyrins, phthalocyanines, or other related heterocycle derivatives (Figure 1)), aiming a specific biological and medical application, especially as antiparasitic agents.

The effectiveness of a photo process depends on several factors including the type of parasite as well as the type and structure of photosensitizers and the reaction medium. This is the consequence of the fact that the action tends to be strongly dependent on biodistribution and cytolocalization, considering their quite local effect, according to the high reactivity and short lifetime of reactive oxygen species such as singlet oxygen and hydroxyl radical or the photosensitizer itself in electronic excited states. Fortunately, it is relatively straightforward to demonstrate selectivity, given the biological staining capacity exhibited by most photosensitizers [43,102,103,104,105,106].

### 3.1. Porphyrins

**Figure 2:**
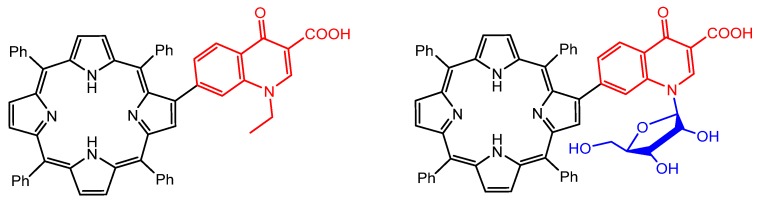
Free-base porphyrin/quinolone conjugates with high singlet oxygen production efficiency, produced by Gomes et al. [107].

The modification with specific groups attached in the *meso* or in the β-positions of the porphyrin ring is widely used to obtain compounds with potential application as antiparasitic agents of great interest. In 2011, Gomes and co-authors reported the synthesis of porphyrins containing quinolones linked to the β-pyrrolic positions, through Suzuki–Miyaura cross-coupling reactions (Figure 2) [107]. These porphyrins showed high singlet oxygen production efficiency, being potential candidates for use against cutaneous leishmaniasis.

**Figure 3:**
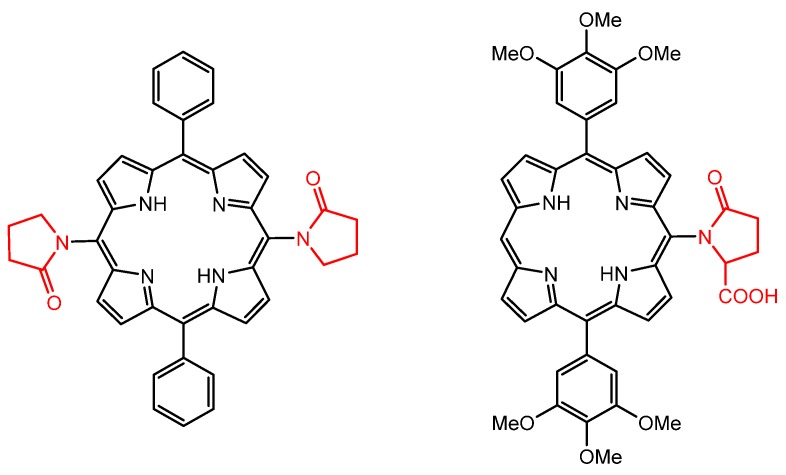
Structures of antiparasitic free-base porphyrin derivatives containing pyrrolidinone moieties, proposed by Abada et al. [51].

In 2013, Abada et al. developed new *meso*-substituted porphyrins containing pyrrolidinone units through Buchwald–Hartwig amination reactions to assess antiparasitic activity against *Leishmania donovani*, *Trypanosoma brucei,* and *Plasmodium sp* (Figure 3) [51].

**Figure 4:**
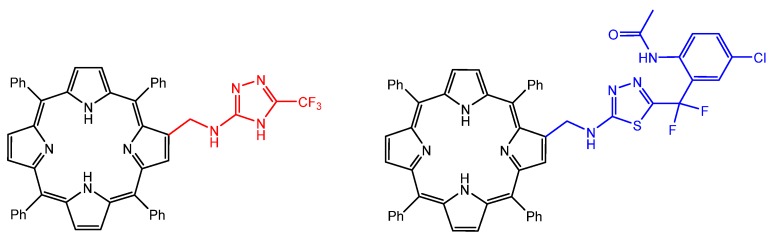
Structures of the amino-triazole and amino-thiadiazole free-base porphyrins with enhanced antiparasitic activity, prepared by Bastos et al. [108].

In the same year, Bastos et al. reported β-substituted porphyrins containing amino-triazole or amino-thiadiazole units, obtained by formation of Schiff bases connecting a β-formylporphyrin and an amino-heterocycle derivative (Figure 4) [108]. The conjugates presented better singlet oxygen production properties and higher values of relative affinity to the leishmanial arginase enzyme, thus improving the antiparasitic activity.

**Figure 5:**
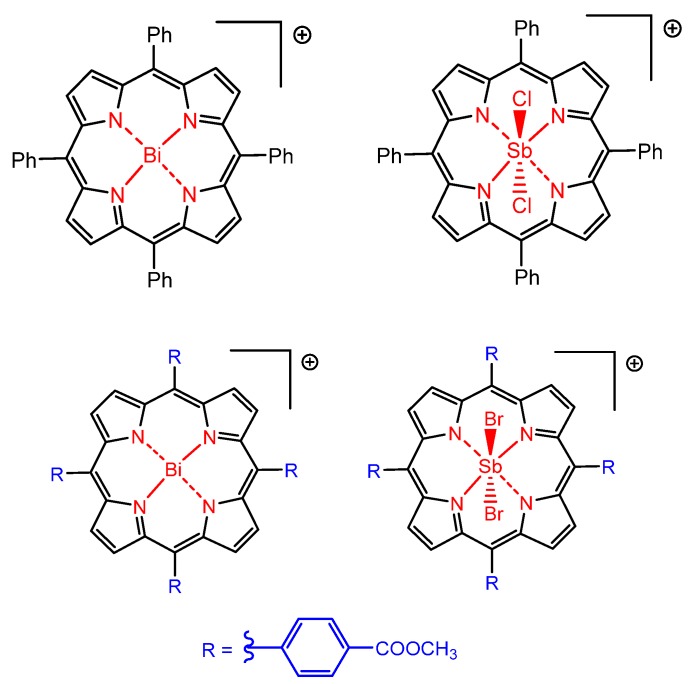
Molecular structures of the Bi(III) and Sb(V) porphyrin derivatives with activity against *Leishmania amazonensis*, by Gomes et al. [43].

Metalloporphyrins also have shown satisfactory activity against parasites, especially bismuth(III) and antimony(V) porphyrins. Gomes and co-authors reported in 2015 the use of porphyrins coordinated with Bi(III) and Sb(V) as active species against the antimony-resistant parasite *Leishmania amazonensis* (Figure 5), where the Sb(V) derivative demonstrated higher antileishmanial activity [43]. The reasons are not clear yet since a fine balance on the lipophilic/hydrophilic character is necessary to allow the dispersion and concentration of those photosensitizers on the target parasites in generally diverse and quite complex and hostile natural environments in which they tend to be used, while keeping their photoactivity. In fact, more research is needed to improve the understanding on the reasons why, for example, the efficacy of Sb(V) derivative is larger than of the respective Bi(III) derivative just based on the above mentioned factors. One possible good reason may be the lower π-stacking tendency of the first one due to the presence of two axial chloride ligands, thus enhancing the fraction of more photochemically active non-aggregated molecules available, or it may be related to a more prosaic factor such as the fact that some first-line drugs for treatment of leishmaniasis are antimonium derivatives such as antimoniate meglumine (Glucantime^®^) and sodium stibogluconate (Pentostam^®^) [109].

**Figure 6:**
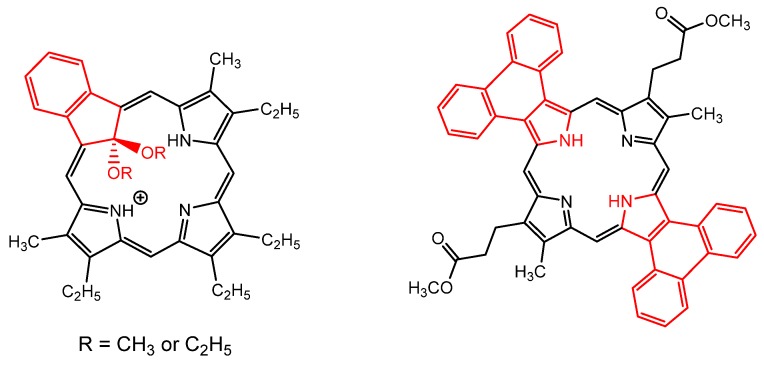
Molecular structures of some porphyrin derivatives tested against *Leishmania* parasitic disease, by Lash et al. [110,111].

Other porphyrin derivatives, such as carbaporphyrins and acenaphthoporphyrins, have demonstrated to be efficient in antimicrobial photodynamic applications by killing parasites such as *Leishmania*. Lash et al. reported the use of alkyl-carbaporphyrin ketal derivatives (Figure 6) for growth inhibition of *Leishmania tarentolae* promastigotes by reactive oxygen species generation, such as singlet oxygen and superoxide [110]. Acenaphthoporphyrin derivatives were incorporated into liposomes, due to their low hydrophilicity, thus increasing their bioavailability and photodynamic effects in vitro against axenic and intracellular amastigotes of the pathogenic *Leishmania panamensis* [111].

**Figure 7:**
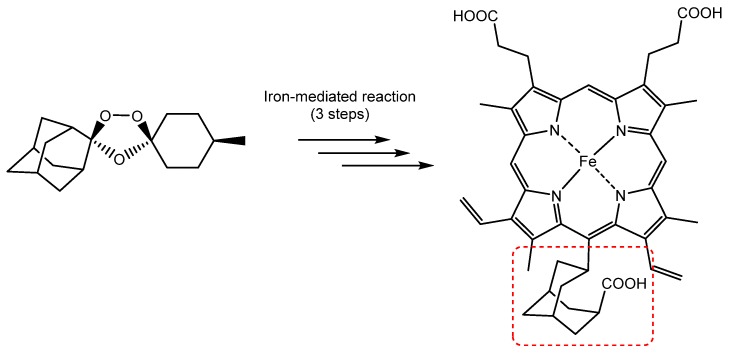
Scheme showing the iron-mediated reaction mechanism of ozonide antimalarials with heme forming ozonide-heme adduct, as demonstrated by the detection of the alkylated product, by Giannangelo et al. [102].

The improved solubility consequence of the incorporation of polar groups (e.g., -OH, -COOH, -OMe) to the porphyrin ring periphery tends to enhance their activity against several parasites. In this context, natural porphyrins analogues, such as those derived from protoporphyrin IX and *mesoporphyrins* (as monomers or dimers), are commonly used as antimicrobial agents against malaria and leishmaniasis parasites [112,113,114,115]. In this way, Giannangelo et al. provided direct evidence that ozonides can react, forming a covalent bond with heme within *P. falciparum*. The formation mechanism of ozonide−heme adducts was demonstrated by detection of the corresponding alkylated product (adamantane derivative) by LC-MS chromatography technique [102] after iron-mediated reaction of heme with the adamantane radical produced by the ozonide (Figure 7).

**Figure 8:**
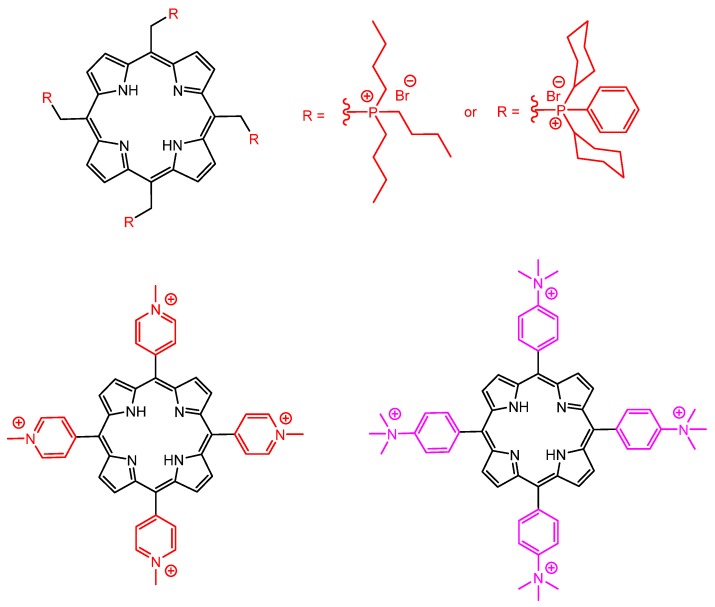
Molecular structures of tetra-cationic porphyrin derivatives used against *Leishmania major* promastigotes, reported by Bristowa et al. [116].

The presence of electrically charged substituents at the periphery of porphyrins also seems to be fundamental to increase the antiparasitic activity. Accordingly, cationic and anionic water-soluble porphyrins probably are the most commonly used molecules for this type of application. Focusing on cationic porphyrins, Bristow et al. reported in 2006 the use of four *meso*-tetra-cationic porphyrins (Figure 8) capable of inactivating *Leishmania major* promastigotes and macrophages infected with *Leishmania,* as well as the promastigote form of the microorganism, by antimicrobial photodynamic therapy techniques [116].

**Figure 9:**
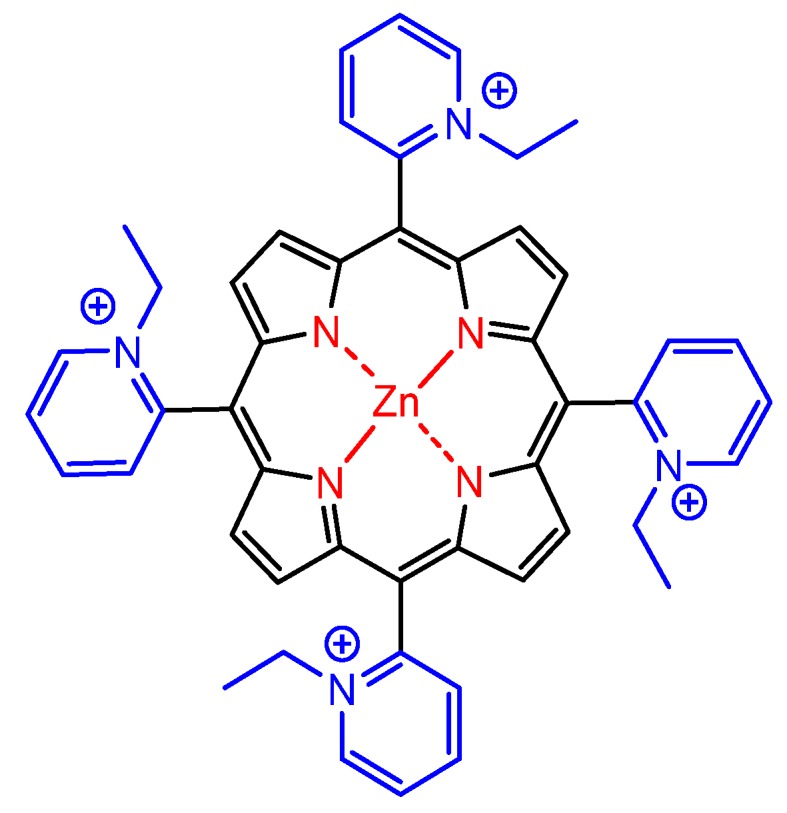
Molecular structure of zinc(II) tetra-cationic porphyrin demonstrating effective antimicrobial photodynamic (aPDT) activity against *Leishmania braziliensis*, by Andrade et al. [42].

Similarly, Andrade et al. used successfully a water-soluble tetra-cationic zinc(II) *meso*-tetrakis(*N*-ethylpyridinium-2-yl)porphyrin (Figure 9) as photosensitizer in the photodynamic treatment of *Leishmania braziliensis* and monitored the results by flow cytometry, optical microscopy, and methyl-thiazol-tetrazolium (MTT) colorimetric analysis [42]. The results indicated that aPDT associated with that tetra-cationic zinc(II) porphyrin represents a promising alternative for treatment of cutaneous leishmaniasis.

**Figure 10:**
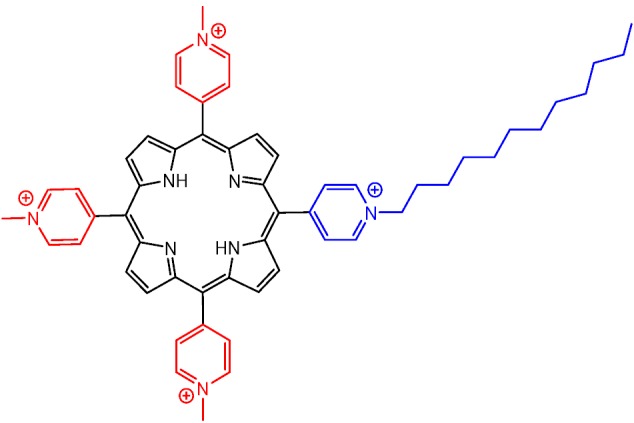
Molecular structure of an amphiphilic water-soluble tetra-cationic porphyrin with good activity against malaria parasites, by Stallivieri et al. [54].

Additionally, amphiphilic photosensitizers have demonstrated enhanced PDT activity since they are more readily internalized by cells and tend to accumulate and damage key cellular organelles, especially the negatively charged mitochondria. The incorporation of a long alkyl chain in tetra-cationic porphyrins tends to keep them soluble in aqueous media, while increasing the capability to interact and be incorporated by biological systems including microorganisms. Accordingly, Stallivieri et al. reported in 2015 the preparation and use of [5-(4-*N*-dodecylpyridyl)-10,15,20-tri(4-*N*-methylpyridyl)porphyrin]tetraiodide, an asymmetric water-soluble cationic porphyrin with high singlet oxygen quantum yield, as a potential PDT agent against malaria parasites (Figure 10) [54].

### 3.2. Phthalocyanines

Phthalocyanines (Pcs) are intensely colored porphyrin-related aromatic macrocycles exhibiting high photochemical activity and potential as photosensitizers for application in photodynamic processes, especially regarding the photo-inactivation of microorganisms and parasites. In contrast with most porphyrin derivatives, this class of molecules has a greater absorption in the red region of the spectrum (in the so called “therapeutic window”) thus favoring the penetration of light. This characteristic allied to their much higher photostability, increasing the perspectives of application since they allow the formation of reactive oxygen species (ROS) deeper into the tissues and for longer periods of time [117,118]. However, phthalocyanines are quite insoluble in most organic solvents and in aqueous media. In general, to overcome this problem, the macrocyclic ring of Pc is modified by incorporation of bulky groups (tert-butyl, pentafluorophenyl, among others) or charged substituents (cationic or anionic Pcs).

Few are the reports describing the use of phthalocyanines for the treatment of parasitic diseases such as malaria and cutaneous leishmaniasis. The first work was reported in 1996 by Lustigmaannd et al. [119], who tested two commercial Si(IV) phthalocyanines (Pc4 and Pc5) against *P. falciparum*. As expected, phthalocyanine Pc4 showed greater activity and toxicity against the malaria parasite when excited with red light. Soon after, Zhao and co-authors reported the use of Pc4-derived silicon(IV) phthalocyanines for sterilization of blood [103] against the malaria parasite. Again, phthalocyanine Pc4 was more active than the other derivatives.

**Figure 11:**
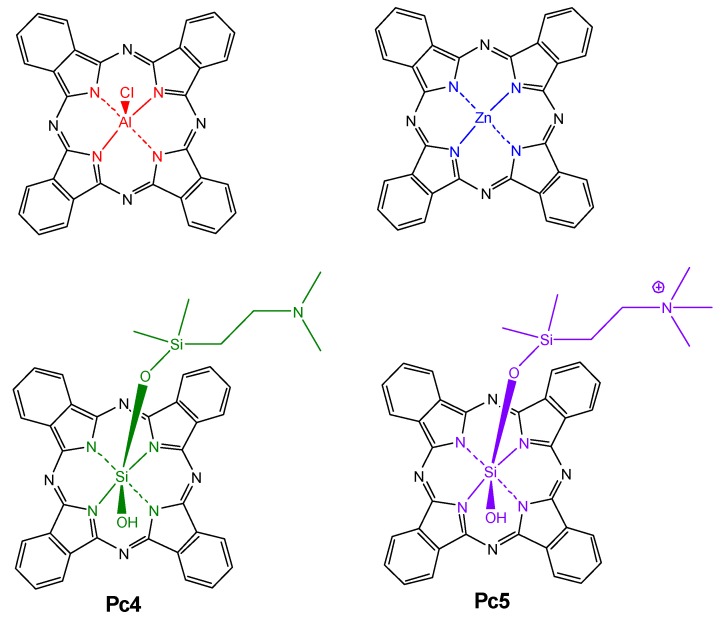
Molecular structures of the Al(III), Zn(II), and Si(IV) phthalocyanines used against *Leishmania amazonensis* by Zhao, Dutta, Escobar, et al. [103,120,121].

Al(III) and Zn(II) phthalocyanines (Figure 11) were reported by Escobar et al. as photosensitizers for inactivation of *L. amazonensis* and other *Leishmania* promastigotes under red-light irradiation. Such unsubstituted phthalocyanines are highly insoluble but, interestingly, showed quite high photodynamic activity against those parasites [120,121].

**Figure 12:**
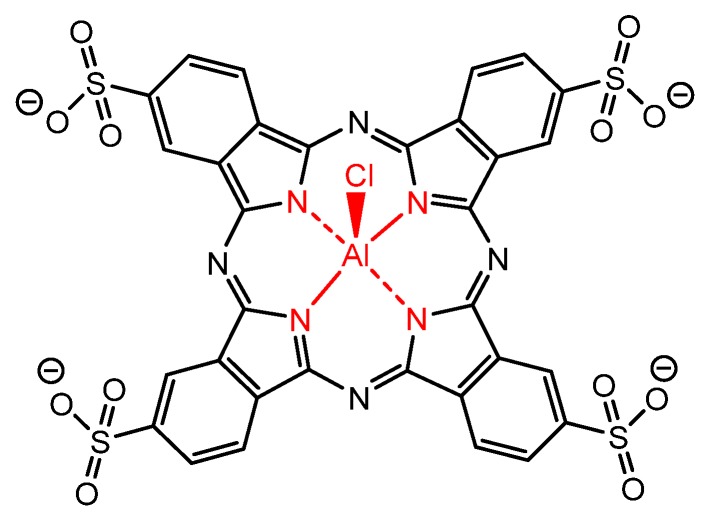
Molecular structure of the tetra-anionic aluminum phthalocyanine presenting good antiparasitic activity against *Leishmania major* and *Leishmania braziliensis*, by Pinto et al. [122].

More recently, in 2011, Pinto et al. reported the photodynamic properties of a water-soluble phthalocyanine (aluminum(III) phthalocyanine tetrasulfonate—AlPcS_4_), against *L. major* and *L. braziliensis*, showing high activity as translated by the high index of mortality of parasites [122] (Figure 12).

### 3.3. Expanded Porphyrins

**Figure 13:**
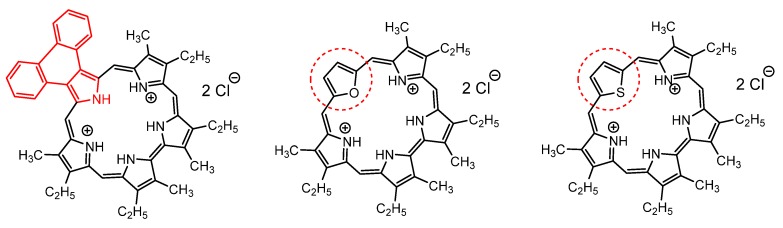
Molecular structures of some representative sapphyrin and heterosapphyrins tested against *Leishmania tarentolae* and *Leishmania panamensis*, by Hooker et al. [104].

Among the expanded porphyrins, sapphyrins (pentaphyrins) are the only related macrocycle whose antiparasitic photodynamic activity was tested to date. In 2012, Hooker et al. reported a study of sapphyrin and heterosapphyrin derivatives (Figure 13) as aPDT agents against *L. tarentolae* or *L. panamensis* amastigotes and promastigotes. In this work, a sapphyrin and two related heterosapphyrins were shown to be particularly effective as inhibitors of *Leishmania* by a mechanism involving the photoinduced generation of reactive oxygen species [104].

## 4. Formulation Techniques

**Figure 14:**
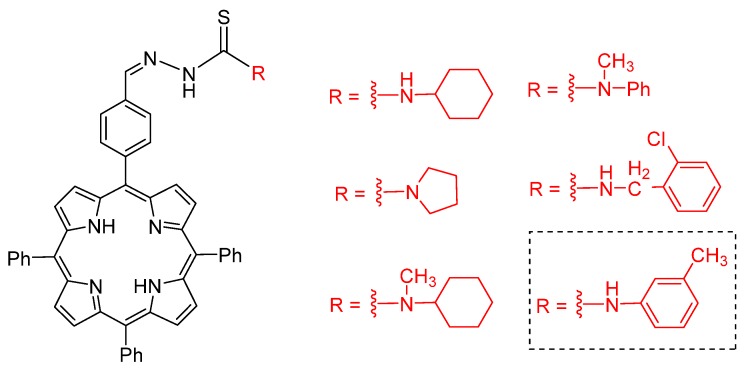
Molecular structures of porphyrin thiosemicarbazides tested against *P. falciparum* by Bhat et al. [123].

As mentioned previously, porphyrins and related derivatives are compounds with a potential antiparasitic and antimicrobial activity. For example, several authors reported the antimalarial aPDT activity against *Plasmodium* parasites [49,50,51,52,53]. Because of their structural similarity with hematin, porphyrins can prevent the formation of Fe(III)-propionate bonds, the critical step in the hemozoin crystal formation [50,56,57]. Structural factors and the presence of central metal ion were reported to be determinant for antimalarial activity as well, as described by Bhat et al. [123] and Cole et al. [124]. Among six free-base porphyrins (Figure 14), it was demonstrated that the one with the lowest steric hindrance on the ring (highlighted in Figure 14) presented higher antimalarial activity when compared to conventional antimalarial drugs. The compounds favor π–π interactions and stacking, leading to the growth of hemozoin crystals [123]. Furthermore, Mg(II), Zn(II), and Sn(IV) porphyrins were more effective in preventing hemozoin formation and were more efficient than the free-base protoporphyrin IX and chloroquine [124].

**Figure 15:**
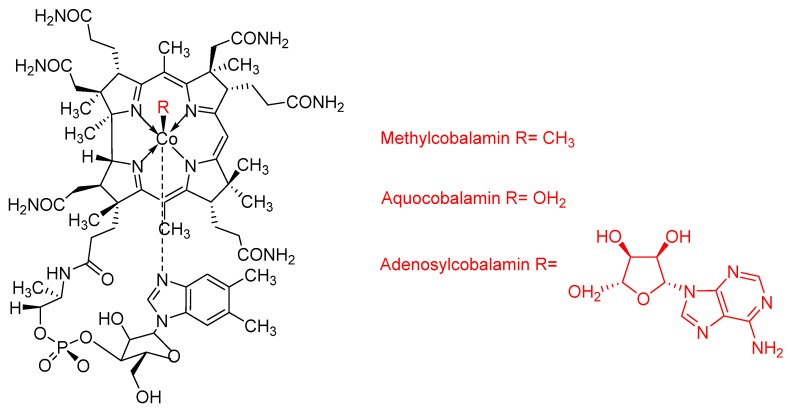
Molecular structures of cobalamins synthetized by Chemaly et al. [53] that were 40 times more effective than chloroquine in inhibiting β-hematin formation in vitro.

Chemaly et al. [53] demonstrated that the vitamin B_12_ adenosylcobalamin (Ado-cbl), methyl-cobalamin (CH_3_-cbl), and aquo-cobalamin (H_2_O-cbl) (Figure 15) were 40 times more effective than chloroquine in inhibiting β-hematin formation in vitro. The mechanism of action of cobalamins is by π–π interaction with the Fe(III)-protoporphyrin IX and also by hydrogen-bonding through their 5,6-dimethylbenzimidazole/ribose/sugar side-chain, decreasing the affinity of other hematin molecules and thus blocking the crystal growth process. The inclusion of targeting agents to the porphyrin macrocycle also can be explored as a strategy to increase their efficiency and selectivity. For example, Marginedas-Freixa et al. [125] demonstrated that the inclusion of the translocator protein TSPO to Zn-protoporphyrin-IX (ZnPPIX) could increase the uptake of porphyrins through the formation of a TSPO2-VDAC complex, leading to accumulation of soluble hemin and, consequently, of reactive oxygen species inside infected red blood cells (iRBC). These toxic species can inhibit growth or induce death of *P. falciparum* parasite. Although metalloporphyrins have shown potential as antimalarials, their slow diffusion through the erythrocyte membrane and difficulties in accessing the parasite food vacuole have limited their efficiency in reducing the growth of *P. falciparum* [52,53]. In this respect, nanosystems can provide novel strategies to deliver those photosensitizers. Some of them have structures similar to biological membranes, allowing them to be transported across the cellular membrane and delivery inside cells [126]. In fact, nanotechnological strategies have been employed to prepare formulations of several medications with increased pharmaceutical efficacy, and, consequently, safety.

Nanocarrier systems can be used to formulate and vehiculate lipophilic chemotherapeutic agents [127] while improving their selectivity and therapeutic activity in vitro and in vivo [128,129,130,131]. Other benefits of nanoencapsulation include advantages such as (1) protection from degradation, (2) longer circulation time in the bloodstream, (3) enhanced bioavailability and cellular uptake, (4) vectorization to selected tissues, (5) prevention of efflux by multidrug resistance pumps, and (6) controlled release [132,133]. Altogether, these benefits minimize the side effects, improve patient comfort, and, consequently, increase adherence of patients to the treatment [20,134]. Furthermore, alternative routes of administration can be made viable (oral, transdermal, etc.) by nanocarrier formulations, for example, facilitating the transposition of biological barriers where conventional drugs cannot reach, such as the blood–brain barrier (BBB) [135,136].

Nanoformulations have also been investigated for malaria treatment. For this purpose, the activity of conventional antimalarial drugs has been enhanced, especially by encapsulation in liposomes, micelles, polymeric particles, cyclodextrin, and dendrimers [137,138,139,140,141,142]. The first work on the incorporation of traditional antimalarial drugs into liposomes was published in 1989 by Peeters et al. [143,144]. They treated a (*Plasmodium berghei*) *P. berghei*-infected mice with liposomal formulation of chloroquine and compared the results with the free drug. The liposomal approach resulted in prolonged availability and significantly higher therapeutic and prophylactic action. A multilamellar nanoliposome was recently described by Fotoran et al. [145] for encapsulation of hydrophilic and lipophilic antimalarial drugs such as chloroquine and artemisinin, respectively. The authors reported IC_50_ values 72% and 60% lower than the free drugs for encapsulated chloroquine and artemisinin, and demonstrated that nanoliposomes interacted more selectively with parasite-infected erythrocytes than with normal red blood cells. PEGylated and non-PEGylated liposomes were prepared by Wang et al. [146] to verify the effect of the polyol on the efficacy of the antimalarial agent febrifugine hydrochloride. PEGylated liposomes showed a more sustained release of the drug and a superior antimalarial activity in vitro. Also, the PEGylated liposomes enhanced the in vivo antimalarial effect in *P. berghei*-infected mice, delaying the recrudescence of the disease and prolonging the survival time, as compared with the free drug and the correspondent conventional non-PEGylated liposomal formulation.

Micelles are also promising systems to improve the bioavailability and solubility of drugs. They can be made of surfactants and polymers, allowing the adsorption of hydrophilic drugs on their surface and the incorporation of lipophilic and amphiphilic substances in their core and shell, respectively [147,148,149]. In this context, more and more complex oligomeric and polymeric units are being prepared to enhance the biocompatibility and loading properties as well as the circulation time by avoiding filtration in the kidneys and recognition by immune system and clearance. For example, recently Coma-Cros [150] reported micellar carriers based on hybrid dendritic-linear-dendritic block copolymers based on Pluronic^®^ F127 and dendrons made of amino-terminated 2,2′-bis(glycyloxymethyl)propionic acid, with a poly(ester amide) skeleton (HDLDBC-bGMPA) connected to an amino-terminated dendronized hyperbranched polymer with a polyester skeleton derived from 2,2′-bis(hydroxymethyl)propionic acid (DHP-bMPA). Chloroquine, primaquine, and quinacrine were loaded into those biocompatible nanosystems that presented drug loading capacity from 30% to 60% and were tested as antimalarials. The dendritic micelles demonstrated high selectivity for *Plasmodium* iRBC as well as capacity to enter those iRBCs and efficiently deliver the drugs thus inhibiting in vitro and in vivo the growth of the *Plasmodium* parasite.

More recently, metal and metal oxide nanoparticles have also been explored as alternatives for antimalarial formulations. An interesting preclinical study using an artesunate formulation with iron oxide nanoparticles was reported by Kannan et al. [151]. In this work, the nanosystem was designed for the sustained pH-dependent release of Fe^2+^ ions within the parasitic food vacuole to induce a toxic ROS spurt. Interestingly, the in vitro growth of *P. falciparum* was retarded at a dose corresponding to just one-eighth of the free artesunate concentration, due to significant cell damage as a consequence of the high concentrations of ROS. Reduction in parasitemia was also reported in vivo after administration of one-eighth to one-tenth of the free drug dosage, demonstrating the superior effectiveness of the formulation prepared with iron oxide nanoparticles.

**Figure 16:**
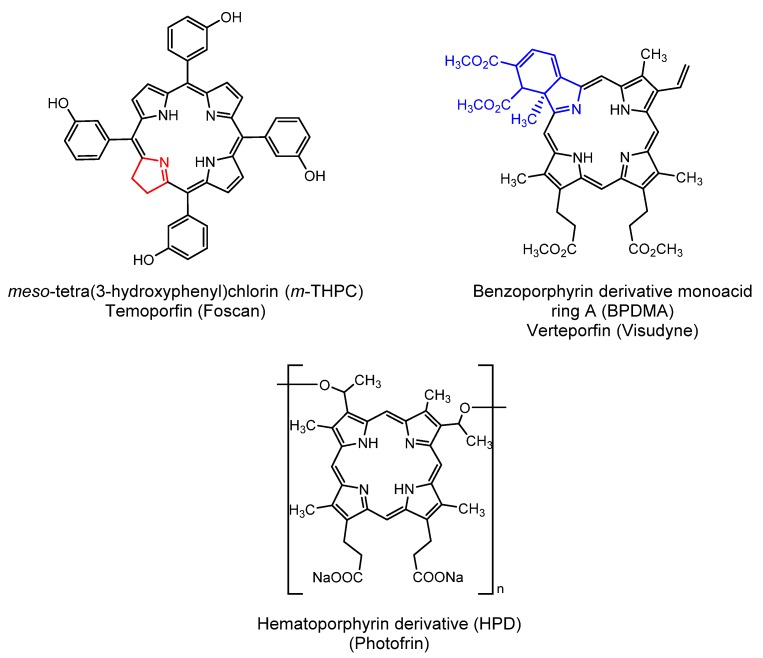
Molecular structures of photosensitizers approved for PDT treatment.

Polymeric nanoparticles are another attractive vehicle for the administration of antimalarials. They can be prepared using a large variety of biodegradable and biocompatible polymers, with different characteristics of resistance and permeability, generating nanoparticles capable of carrying and modulating the release of drugs while protecting them from the medium, pH, light, and enzymatic degradation [152,153,154,155]. Polymeric nanocapsules have presented promising results for administration of antimalarials, improving their efficacy, as demonstrated in several in vivo studies [156,157,158,159,160]. For example, polymeric polyvinylpyrrolidone 10 (PVP 10), l-α-phosphatidylcholine, polysorbate 80, and Poloxamer 407 nanoparticles were used to encapsulate decoquinate (DQ), a new antimalarial candidate. The efficacy of the encapsulated DQ formulation was significantly superior, facilitating the suppression of the liver stage and growth of *P. berghei* parasite in infected mice and the growth of *P. falciparum* parasite in the blood stage in vitro [161]. Porphyrin derivatives can also be combined with different micro and nanometric materials, enabling the development of new formulations with the potential to treat several diseases. Covalent conjugation, or physical inclusion of porphyrins to nanoparticle carrier systems, changes the way how they interact with the biological medium. As consequence, the biodistribution and therapeutic efficiency are modified as well while facilitating the dispersion of water-insoluble pharmaceuticals [67]. Several nanoformulations have been developed and evaluated by researchers around the world [162,163,164] and some of them have a high degree of technological maturity or are already commercially available such as Temoporfin (Foscan^®^, Foslip^®^, Fospeg^®^) and Verteporfin (Visudyne^®^) (Figure 16). Both are liposomal formulations of porphyrin derivatives for the treatment, respectively, of cancer and macular degeneration using PDT. Both porphyrin derivatives used as photosensitizers have high hydrophobic character making them prone to precipitation in biological medium in the free form but not as a liposomal formulation [165]. Temoporfin was significantly more effective for tumor treatment due to the enhanced local accumulation by the increased permeability and retention mechanism while reducing the damage to healthy tissues and increasing the circulation time [20,166,167]. Other examples include the porphyrin derivative Photofrin II^®^ (Figure 16). Lamch et al. [168] encapsulated the porphyrin photosensitizer in polymeric micelles prepared with a mixture of Pluronic P123 and F127, and its photodynamic properties against human breast MCF-7/WT (caspase-3 deficient) and ovarian SKOV-3 (resistant to chemotherapy) cancer cell lines were evaluated.

The formulation was biocompatible, showing low cytotoxicity in the dark but high ROS level and enhanced PDT activity against tumor cells under irradiation, as compared to free Photofrin II^®^. This was also formulated with chitosan derivatives generating a nanosystem with reduced fluorescence quantum yield and fluorescence lifetime when compared to free Photofrin^®^, clearly indicating that the incorporation in micelles was suppressing its photoactivity. Interestingly enough, the in vitro results presented much intense fluorescence than free Photofrin^®^, consistent with much stronger phototoxicity, responsible for the significant levels of apoptosis induced in human pancreatic cancer cells [169]. Temizel et al. [170] evaluated the PDT efficacy of two porphyrin formulations against HeLa and AGS cancer cell lines: The protoporphyrin IX functionalized with lipophilic oleylamine arms (PPIX-Ole) in the free form and encapsulated into 1,2-dioleyl-sn-glycero-phosphatidylcholine (DOPC) liposomes. The results showed that both were more photoactive than conventional PPIX, and the degree of toxicity was dependent on the liposomal concentration, efficiency of delivery of the photosensitizer, and cancer cell type.

The porphyrin derivatives’ formulations based on nanocarriers showed a significantly increased photodynamic efficacy since the surface properties of nanostructures can favor their interaction with biological systems and those properties can be modulated to realize more effective nanosystems. For example, Molinari et al. [171] described the enhanced uptake of the hydrophobic chlorin 5,10,15,20-Tetrakis(3-hydroxyphenyl)chlorin (*m*-THPC) and total destruction of various human glioblastoma cell lines (A172, DBTRG, LN229, U118) in vitro after irradiation with light. Bovis et al. [172] investigated the in vivo biodistribution and accumulation of two PEGylated liposomal *m*-THPC formulations (FosPEG 2% and FosPEG 8%), in comparison to standard Foscan^®^. The PEGylated liposomal formulation increased the blood plasma circulation time and enhanced permeability and retention (EPR) effect, as well as tumor selectivity in comparison to Foscan^®^, enhancing the PDT activity.

Polyethylene glycol was also investigated as adjuvant in a formulation to encapsulate zinc phthalocyanine (ZnPc). The lipid concentration in the liposomes was responsible for the modulation of the efficiency of cellular uptake and cell death by PDT-mediated oxidative processes [173]. Another example of modulation of the photodynamic activity by liposomal composition was reported by de Oliveira et al. [174]. They evaluated the photodynamic activity of ZnPc incorporated in unilamellar liposomal formulations containing increasing concentrations of cholesterol that improved the stability of the particles, optimized the release of ZnPc, and modulated its phototoxicity against several human tumor cells. PEGylated poly(d,l-lactide-co-glycolide) nanoparticles (NPs) were employed to encapsulate indium(III) phthalocyanine (InPc) and its PDT efficacy against MCF-7 breast tumor cells was evaluated by Souto et al. [175]. It was demonstrated in this way that other factors than the chemical composition of the polymeric shell, such as the phthalocyanine concentration, incubation time, and laser power, can modulate the therapeutic effects.

**Figure 17:**
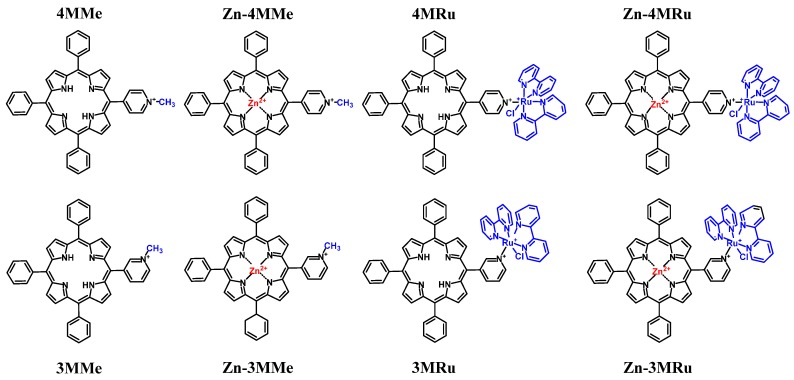
Molecular structures of some hydrophobic porphyrins and metalloporphyrins encapsulated into polymeric nanocapsules by Deda et al. [176,177].

Deda et al. demonstrated that micro- and nanocapsules of marine atelocollagen and xanthane gum are excellent vehicles for delivery of hydrophobic porphyrins and metalloporphyrins (Figure 17). This system was able to penetrate the membrane of tumoral HeLa cells, reaching the cytoplasm where the photosensitizer is released, promoting the destruction of tumor cells upon irradiation with visible light of appropriate wavelength [176,177]. In addition, the encapsulation changed the mechanism of cell death by triggering apoptosis rather than necrosis, the mechanism of death of cells treated with the free porphyrin in solution [126]. Promising results were also reported by Sutoris et al. for a liposomal formulation incorporating hydroxy-aluminum phthalocyanine (AlOH-Pc, a noncommercial porphyrin derivative, for topical PDT treatment of prostate carcinoma [178] and mammalian carcinoma [179]. The first study resulted in 100% cure of the experimental animals, and the second one revealed that the nanoformulation led to complete tumor remission in 90% (9/10) of experimental animals, in contrast with the commercially available Metvix that only postponed the tumor growth.

The conjugation of specific ligands on the surface of nanosystems, including vitamins, glycoproteins, peptides, oligonucleotides, aptamers, and antibodies, has been explored to increase their selectivity by specific target cells and tissues [180]. This strategy has shown to be very promising for treatment of several diseases [181,182,183,184,185], including malaria. More recently, Biosca et al. [186] described an immunoliposomal nanosystem encapsulating the antimalarial drugs pyronaridine and atovaquone. The conjugation of these encapsulated antimalarials with antibodies against glycophorin A, a protein present in red blood cells (RBC), increased the drug targeting and delivery into *Plasmodium* iRBC and gametocytes, the stage responsible for the transmission of the malaria infection from the vertebrate host into mosquito vector. Finally, the encapsulated drugs presented higher activities than their free forms when tested in in vitro *P. falciparum* cultures, resulting in up to 50% inhibition of parasite growth.

**Figure 18:**
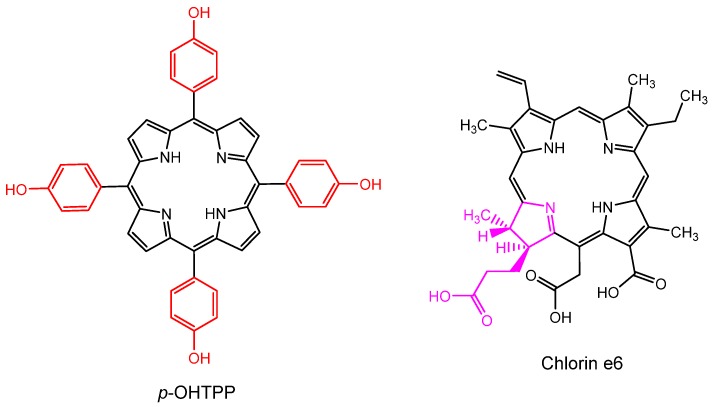
Molecular structures of 5,10,15,20-tetrakis(4-hydroxyphenyl)porphyrin and chlorin e6, which was incorporated into hyaluronic acid-coated liposomes by Feng et al. and Jung et al. [189,190].

The vectorized controlled release of therapeutic agents specifically at tumor sites is one of the best approaches for cancer treatment with reduced or minor side effects. The major strategy for this purpose consists of conjugating suitable ligands on the nanosystem surface able to bind specifically to receptors that are overexpressed by tumor cells or at the tumor vasculature. For example, Liang et al. [187] conjugated folic acid onto polyvinylpyrrolidone (PVP) micelles containing zinc phthalocyanine (ZnPc) in a non-aggregated form, as confirmed by UV-VIS spectroscopy, conferring high photodynamic activity in vitro and in vivo. However, the vectorization capability of folic acid is relatively low, such that other biomolecules, such as aptamers and antibodies, have been reported for that purpose. For example, Abdelghany et al. [188] conjugated an antibody-targeting death receptor 5 (DR5), a cell surface apoptosis-inducing receptor upregulated in various types of cancer cells, on the surface of chitosan/alginate nanoparticles loaded with the hydrophilic photosensitizer *meso*-tetra(*N*-methyl-4-pyridyl)porphyrin tetra-tosylate (TMP). The conjugation of that antibody enhanced the uptake and the therapeutic effect of the TMP on HCT116 cells. The hyaluronic acid (HA) derivative with aldehyde group was recently used by Feng et al. [189] to functionalize liposomes and enhance their uptake and effectiveness against CD44 overexpressing cancer cells. The uptake of hyaluronic acid (HA) conjugated nanosystem was proven to be specific to MDA-MB-231 cells and provided an efficient strategy to enhance the photo-cytotoxicity of 5,10,15,20-tetrakis(4-hydroxyphenyl)porphyrin (*p*-OHTPP) (Figure 18). Hyaluronic acid was also investigated by Jung et al. [190], who synthesized novel types of nanophotosensitizers based on hyperbranched chlorin e6 (Ce6) conjugated with hyaluronic acid (HA) targeting CD44-receptors. The nano-photosensitizers showed higher intracellular accumulation of Ce6 and higher ROS generation and PDT efficacy than free Ce6. The formulations were also selective as confirmed by in vivo tumor xenograft, which demonstrated that the fluorescence intensity in the tumor tissues was much stronger than those in other organs.

More complex nanoformulations can be used to perform combination therapy, whereby two or more therapeutic agents are incorporated in the same particle [186,191,192]. One example for malaria treatment of *P. berghei*-infected mice was based on the administration of a liposomal formulation loaded with curcuminoids in combination with α/β arteether. This strategy showed that combined therapy can cure infected mice and prevent recrudescence [193]. Promising results were also reported by Isacchi et al. [194] that described a reduction of *P. berghei* NK-65-infected murine malaria model parasitemia by administration of PEGylated liposomal formulations loaded with artemisinin and curcumin.

Nanoformulations have also allowed the investigation of combined action of drugs for cancer therapy. An interesting approach was explored by Park et al. [195], who developed a micellar nanosystem (30 nm) on which the amphiphilic units are constituted by Chlorin e6 (Ce6) conjugated to Pluronic F127^®^. Then, the anticancer drug doxorubicin (DOX) was incorporated in the micellar core. The in vitro and in vivo studies on drug-resistant cancer cells demonstrated that the photoinduced generation of ^1^O_2_ causes cellular membrane damage (lipid peroxidation), which enhances the cellular uptake of DOX. This was found to be a fundamental process to overcome the drug resistance in cancer cells without undesirable side effects. This synergic effect was also explored by Yu et al. [196], who encapsulated the chemotherapeutic agent 5-fluorouracil (5FU) in a novel core@shell cross-linked dextran–hemin micelle. Hemin was used as a photo-triggered switch for the controlled release of 5FU, that concomitantly acted as a PDT agent.

**Figure 19:**
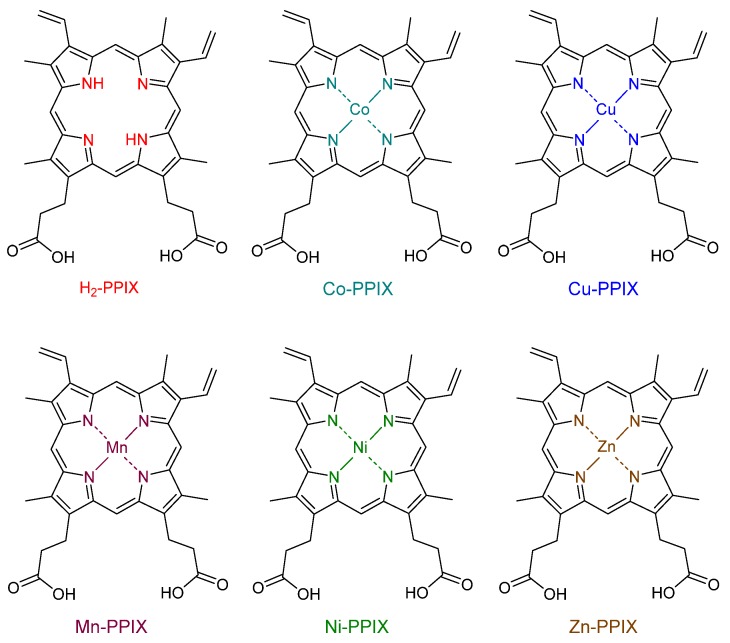
Molecular structures of protoporphyrin IX and the metallated derivatives, which were encapsulated into polymeric nanocapsules and tested against *P. falciparum*. [55].

Although the strategy based on nanoparticles has been extensively investigated to vehiculate several medicines and porphyrin derivatives aiming the treatment of many diseases, it has been less explored in the case of porphyrin-based antimalarial agents. The metallated derivatives of protoporphyrin IX (PPIX), Fe(III)-PPIX, Zn(II)-PPIX, Ni(II)-PPIX, Cu(II)-PPIX, and Co(III)-PPIX (Figure 19) were incorporated into polymeric nanocapsules of marine atelocollagen/xanthan gum, and their antimalarial activity was evaluated on RBCs cultures infected with *P. falciparum* [55]. All nanoformulations presented better antimalarial activity than the respective free porphyrins, indicating that the polymeric nanosystem promotes the internalization of those metalloporphyrins into infected RBC cells, allowing their direct access to the parasitic food vacuoles. ZnPPIX nanoformulation showed slightly larger efficiency, reducing the growth of the hemozoin crystals by 34% when compared to chloroquine (28%) [55].

Differently from malaria parasites, the host does not rely on hemozoin crystal formation for hemin detoxification. The host catalytically degrades hemin into biliverdin (BV) [197]. Nanomolar concentration of BV can delay the intraerythrocytic development of *P. falciparum* by targeting parasite enolase [198]. Moreover, the malaria digestive vacuole (DV), where hemoglobin degradation and hemozoin formation takes place, is also an important compartment for calcium homeostasis [199]. Considering the DV is a site of action of chloroquine and other antimalarials based on porphyrins, any formulation technique that helps the delivery of these drugs into parasite intracellular compartments has the potential to strengthen their pharmacological treatment.

## 5. Applications of Porphyrins as Vector Control and Biosensors

The treatment and immunization targeting the parasite is just one front to deal with diseases that rely on insect vectors to disseminate into population. The photosensitizing properties of hematoporphyrin IX and other porphyrin-based drugs have also been investigated as a new and environmentally friendly approach to induce oxidative damage to mosquitoes’ larvae of gender *Culex* (vector of West Nile virus, Japanese encephalitis, filariasis, and avian malaria disease) [200], *Aedes* (vector of dengue, Zika, yellow fever, and chikungunya virus) [200,201], *Anopheles* (vector for human malaria) [45], and also on adult flies of *Bactrocera oleae* species (olive cultures pest), *Ceratitis capitate* (pest for more than 200 species of fruits and vegetables), and *Stomoxys calcitrans* (pest for cattle) [202]. In all cases, the same concept and strategy of PDT was used to release insecticides based on porphyrins triggered by sunlight. As the photochemical activation of these compounds by intense sunlight occurred on lower doses, they were not considered toxic for the environment. 

The search for more environmentally friendly, low cost, and water-soluble porphyrin-based photosensitizers to control pest populations revealed that chlorophyll derivatives (chlorophyllin and pheophorbide) are the most effective ones against larval stages of several insects including mosquitoes, freshwater snails, and certain parasites of fish [203,204,205]. The photodynamic toxicity of chlorophyll is also supported by field trials, where the addition of up to 100 µM of chlorophyll derivatives in infested swamps and sand pits in Uganda killed 85–100% of *Anopheles gambiae* larvae without affecting other nontargeted organisms such as the larvae of dragon fly and predator mosquitoes [206]. Moreover, chlorophyll derivatives are nontoxic to humans and some of these compounds are even certified as food colorants (identified as E140 and E141 in the European Union) [203]. Fortunately, since the toxic effects of chlorophyll derivatives are triggered by light, they represent low or no harm to nontransparent organisms.

Chlorophyll also has broad application as biosensor for herbicides, pesticides, heavy metals, and other hazardous toxicants widely spread on the environment [207]. Such sensing capability is based on its characteristic fluorescence emission and the role it has on the photosynthetic process machinery that is quite sensitive to damage caused by a variety of physical and chemical stress [207,208]. The practical application of this strategy has provided a wide range of information on cultures resistant to heat, cold, light, drought, and salt stress [209]; to the effect of pesticides, herbicides, and growth regulators on plant [210]; genotypic differences in plant breeding; and the freshness of vegetables, flowers, and fruits [211,212,213].

There are a variety of natural heme-based sensors in biological systems that are used to perceive nitric oxide (NO), O_2_, and CO_2_ and the chemistry required to adapt to the changes in their availabilities in both eukaryotic and prokaryotic organism, as discussed in a thorough review by Rodgers et al. [214]. Such heme-based sensors not only helped the elucidation of many signaling events, biosynthetic formation, and biological activity of natural compounds, but also contributed to the basic knowledge on those new biotechnological approaches to deal with modern problems like the spread of environmental pollutants. Halogenated organic compounds constitute a large group of chemicals generally found in herbicides, insecticides, fungicides, plasticizers, and solvents that are accumulating in the biosphere and creating public concerns due to their toxicity, persistence, and bioconcentration [215]. Enzymatic biodegradation of halogenated compounds relies on transition metal cofactors such as porphyrins and derivatives [216,217,218,219,220] and has been used as tools for environmental mitigation.

Electrodes coated with porphyrin films exhibit electrocatalytic properties and are useful as biomimetic sensors. For example, graphene coated with iron porphyrin can be used for real-time detection of NO in biological samples [221]. Cobalt porphyrin-modified electrodes can be used for rapid screening of toxic organohalides in aqueous samples with no need of pretreatment for removal of oxygen [222]. Carbon fiber coated with Ni(II) (tetrakis-[(3-methoxy-4-hydroxyphenyl)]porphyrin), TMHPP, has also been used to quantify NO and its decay products in biological systems [223,224,225]. These voltammetric sensors based on porphyrins can be used to analyze a variety of compounds depending on the metal ion coordinated to the macrocyclic ring responsible for tuning their electrocatalytic properties. For example: sugars [226], hydrazine [227], phenols [228] and DNA [229] can be detected by Cu(II)-porphyrin analogues; dopamine and neurotransmitters [230] by Zn(II)-porphyrin derivatives; heavy metals and Cu(II) [231] by metal-free-porphyrins; and alcohols [232] by Ni(II)-porphyrin derivatives. From the perspective of coordination chemistry, porphyrins constitute quite versatile macrocyclic ligands able to chelate almost all known metal ions.

Metalloporphyrins have broad catalytic properties since they can interact with several molecular species through their axial positions, as demonstrated by their numerous applications in chemical analysis. Several porphyrins are useful as spectrophotometric reagents for quantitative analyses of a variety of metal ions [233,234,235,236] and as active materials for electroanalysis and electrochemical biosensors. Metalloporphyrins are also used on potentiometric sensors, a process that exploits selective binding events to transduce the analytic ionic activity of a solution into a potential readout. A practical example of potentiometric sensors is the measurement of chloride in human serum samples using Mn(III)-porphyrin derivatives [237].

## 6. Conclusions

Porphyrin, phthalocyanines, and related macrocycles exhibit exciting absorption and photochemical properties leading to the local production of reactive oxygen species, such as singlet oxygen, hydroxyl radical, and peroxide, or the excited photosensitizers can act as strong oxidizing agents promoting cellular damage. Despite such interesting characteristics some drawbacks must be overcome. Among them is the quite low solubility and very strong tendency of π-stacking, generally promoting the disappearance of photosensitizing properties. Another key feature that must be realized is targeting as specifically as possible the diseased tissues/cells, microorganisms, or parasites to decrease the therapeutic dose and avoid side effects or environmental contamination, while maximizing the effectiveness. In fact, nonselective dispersion of the photosensitizer can impair healthy tissues or, worse, may not be able to promote enough damage to kill all tumor cells and avoid recurrence. Thus, it urges finding strategies to promote the accumulation of as large as possible amounts of the porphyrin derivatives in key organelles or organs by active targeting strategies. This can be achieved by tailoring the structure of porphyrin derivatives, exploiting their rich and versatile chemistry, and combining with nanotechnology tools to enhance bioavailability and provide effective targeted delivery and improved effectiveness. This alternative can include the combined action of known drugs with new drugs based on porphyrin derivatives to get additive or synergic effects in biomedical applications and as antiparasitic agents. In fact, these are not exclusive of PDT but key issues that will boost the development of molecularly enabled personalized precision diagnosis and treatment. Accordingly, the possibilities of application of porphyrin derivatives are vast and increasing, fitting with the multiple roles of these compounds in nature, opening broad and auspicious perspectives for the development of new materials and treatment agents. This is not a surprise considering the architectural flexibility and facile chemical tailoring, allowing the preparation of a multitude of derivatives with suitable bioavailability, interaction with biological systems, and photochemical and luminescence properties, depending on the metal ion coordinated to, as well, as the substituents attached to the macrocylic ring.
